# The evolution of reproductive isolation in *Daphnia*

**DOI:** 10.1186/s12862-019-1542-9

**Published:** 2019-11-27

**Authors:** Tiffany A. Chin, Carla E. Cáceres, Melania E. Cristescu

**Affiliations:** 1 Department of Biology, McGill University, 1205 ave Docteur Penfield, Montreal, Quebec, H3A 1B1 Canada; 2 School of Integrative Biology, University of Illinois at Urbana-Champaign, Urbana, IL 61801 USA

**Keywords:** *Daphnia pulex*, Gene flow, Genetic incompatibilities, Postzygotic isolation, Prezygotic isolation, Speciation

## Abstract

**Background:**

The process by which populations evolve to become new species involves the emergence of various reproductive isolating barriers (RIB). Despite major advancements in understanding this complex process, very little is known about the order in which RIBs evolve or their relative contribution to the total restriction of gene flow during various stages of speciation. This is mainly due to the difficulties of studying reproductive isolation during the early stages of species formation. This study examines ecological and non-ecological RIB within and between *Daphnia pulex* and *Daphnia pulicaria,* two recently diverged species that inhabit distinct habitats and exhibit an unusual level of intraspecific genetic subdivision.

**Results:**

We find that while ecological prezygotic barriers are close to completion, none of the non-ecological barriers can restrict gene flow between *D. pulex* and *D. pulicaria* completely when acting alone. Surprisingly, we also identified high levels of postzygotic reproductive isolation in ‘conspecific’ interpopulation crosses of *D. pulex*.

**Conclusions:**

While the ecological prezygotic barriers are prevalent during the mature stages of speciation, non-ecological barriers likely dominated the early stages of speciation. This finding indicates the importance of studying the very early stages of speciation and suggests the contribution of postzygotic isolation in initiating the process of speciation.

## Background

The process of speciation often involves the emergence of multiple reproductive isolating barriers (RIB) that obstruct gene flow between sister species [1–3]. Over the last two decades, several important studies focused on the interplay between early acting barriers (prezygotic barriers that reduce the probability of zygote formation) and late acting barriers (postzygotic barriers). Studies examining the absolute and relative contributions of multiple RIB suggest that prezygotic isolating barriers have a larger impact in reducing gene flow between species compared to postzygotic isolating barriers [4–7]. However, when prezygotic barriers are permeable, and incipient species come in contact, postzygotic barriers can also play an important role in keeping gene pools distinct [8–10], either directly at a high reproductive cost of hybrid inviability and infertility, or indirectly by driving the reinforcement of prezygotic reproductive barriers.

Despite major advancements in our understanding of the speciation process, the contribution of various reproductive isolating barriers (RIB) in restricting gene flow and the sequential order in which these barriers emerge during the process of speciation remains poorly understood [11, 12]. Few notable studies examine the accumulation of RIB of closely related species pairs of *Drosophila* [13], fish [14–16], and plants [17, 18] at various stages of the speciation continuum. These comparative studies show that prezygotic isolating barriers can evolve quickly in comparison to postzygotic isolating barriers, and that multiple barriers often accumulate during the process of speciation [19]. Thus, it has been assumed that prezygotic isolating barriers emerge earlier to postzygotic isolating barriers and might be more important during the onset of speciation. Moreover, current approaches to estimate the relative strengths of reproductive isolating barriers order barriers according to the life history stages of an organism, due to the observation that these barriers act sequentially to restrict gene flow [4, 20]. As such, early acting prezygotic barriers often show greater relative contributions to reproductive isolation when compared to late acting postzygotic isolating barriers [2, 4]. However, in some cases, postzygotic isolating barriers can emerge before prezygotic isolating barriers [9, 21, 22], particularly during the early stages of speciation [23, 24], and can also evolve relatively fast [18, 25, 26]. Other studies point to prezygotic and postzygotic isolating barriers evolving at similar rates [27]. Collectively, these studies highlight the need for more empirical research on the emergence of RIB at various stages of the speciation continuum with particular attention on the early stages of speciation that are much more difficult to investigate [28].

Ecological prezygotic isolating barriers such as habitat and temporal isolation can arise as a by-product to populations adapting to different environments [29–31]. Nonecological prezygotic isolating barriers such as behavioural isolation, expressed as differences in mating rituals and behaviours, may also evolve independently from or in concert with ecological barriers or reproduction [3, 32]. Additionally, postzygotic isolating barriers (intrinsic or extrinsic) can come into effect when incipient species come into contact [8, 33]. Often, speciation is considered a long process, with RIBs continuing to evolve and accumulate even after the cessation of gene flow [3, 12]. Thus, studies on mature species pairs that are approaching the completion of the speciation process cannot be used efficiently to infer the RIBs involved during the early stages of the speciation. Unfortunately, few studies contrast the RIBs involved in the early and late stages of speciation. The few studies that conduct empirical RIB studies at the intra- and inter-specific level, point to the importance of postzygotic isolating barriers at restricting gene flow during the early stages of speciation among lineages in the rainwater killifish *Lucania parva* [24], the spring peeper chorus frog *Pseudacris crucifer* [34], and the copepod *Tigriopus californicus* [35]. However, studies on *Drosophila melanogaster* show that prezygotic isolating barriers can emerge under artificial selection for body sizes [36]. Thus, the early stages of the speciation process continues to be poorly understood.

The *Daphnia pulex* species complex is an ideal system to study the processes of speciation from early stage to the more mature stages of the speciation. It consists of 12 genetically distinct lineages with various degrees of reproductive isolation [37, 38], with several lineages exhibiting high ‘intraspecific’ genetic structure across small spatial scales [39–41]. Two morphologically similar species in this complex, *Daphnia pulex* (Leydig) and *Daphnia pulicaria* (Forbes), are widely distributed across North America (Additional file 2: Figure S1) and are thought to be in the process of speciation [41]. Divergence between *D. pulex* and *D. pulicaria* occurred relatively recently, with an estimate of less than 2 mya according to mitochondrial markers [37], and about 82 kya according to nuclear markers [42]. Due to their morphological similarities, and the ease with which crosses can be conducted under laboratory conditions, the status of the two species has been highly debated [41, 43–47]. The two species primarily inhabit distinct habitats, with *D. pulicaria* occurring in permanent stratified lakes, and *D. pulex* inhabiting ephemeral, fishless ponds [48, 49]. These habitats provide a variety of selective pressures, shaping interspecific differences in life history traits [50–53]. For example, predatory responses differ between the two species as *D. pulex* avoid invertebrate predation (e.g., *Chaoborus)* by producing neck-teeth and hardened carapace [54], while *D. pulicaria* use vertical migration to avoid fish and invertebrate predation [55].

Habitat segregation is considered to play an important role in restricting gene flow in these ecological species. However, the two species can often come in contact due to flooding events, bird migration, and anthropogenic disturbances. Laboratory F1 hybrids of *D. pulex* females crossed with *D. pulicaria* males have been successfully constructed in the past [56], yet true F1 hybrids with cyclical parthenogenetic reproduction are rarely found to occur naturally, either due to the presence of ecological or non-ecological isolating barriers that play an important role in the speciation process of *Daphnia*. Detailed genetic studies confirm strong habitat segregation while also revealing unexpectedly high levels of intraspecific genetic structure occurring at low geographic scale within these ecological species [39, 57].

In this study, we estimate and compare ecological and non-ecological barriers between and within the two closely related species: *D. pulex* and *D. pulicaria*. We conduct bidirectional no choice crosses to determine the absolute and relative contributions of RIB for early-acting (mating-fertilization) and late-acting (F1 zygotic mortality, F1 hatching success, F1 survivorship) isolating barriers to speciation. We quantify these reproductive isolating barriers and compare RIB strengths in sympatry and allopatry. Additionally, we investigate the degree of genetic cohesion across large geographic distances to determine whether emerging speciation is occurring within each of these two species. We discuss our results in the context of the evolutionary forces that shape both early-acting and late-acting RIBs.

## Results

### Non-ecological prezygotic isolating barrier

We constructed a total of 504 no-choice crosses from individuals sampled in pond and lake habitats (Table 1; Additional file 1: Table S1)*,* 274 conspecific crosses for *D. pulex* (px x px) and *D. pulicaria* (pc x pc) and 230 heterospecific crosses (pc_♀_ x px_♂_; px_♀_ x pc_♂_; Additional File 1: Table 2). Here we discuss our results with respect to the absolute and relative contributions of non-ecological reproductive isolating barriers acting between *D. pulex* and *D. pulicaria*.

**Table 1:** Habitat locations, reproductive mode (RM) and molecular identification of *Daphnia pulex* and *Daphnia pulicaria* used in this study. All individuals (n) were found to be cyclically parthenogenic (CP) using methods from [58]. Molecular identification based on the mitochondrial (mtDNA) marker NADH dehydrogenase subunit 5 (ND5) allowed us to assign clade membership as in [59]. The nuclear (nDNA) *lactate dehydrogenase* A (LDH) locus was used to identify individuals that were homozygous for the F allele (lake phenotype) or the S allele (pond phenotype), or were heterozygous for both alleles (hybrid phenotype)

						mtDNA	nDNA
Location	Clone ID	Lat	Lon	n	RM	ND5 clade	LDH
*Ponds*							
Center, IL	CEN	40.13	−88.14	3	CP	Panarctic *D. pulex*	SS
Dump, IL	DUM	40.24	−87.78	2	CP	Panarctic *D. pulex*	SS
Bridge North, IL	BRI	40.12	−87.74	2	CP	Panarctic *D. pulex*	SS
Top, IL	TOP	40.24	−87.78	2	CP	Panarctic *D. pulex*	SS
Disputed, ON	DIS	42.17	−83.03	3	CP	Panarctic *D. pulex*	SS
Solomon, MI	SOL	42.71	−84.38	2	CP	Panarctic *D. pulex*	SS
St. Michael, ON	STM	42.23	−83.07	2	CP	Panarctic *D. pulex*	SS
*Lakes*							
Clear, IL	CLE	40.14	−87.74	2	CP	Panarctic *D. pulex*	FF
Deep, IL	DEE	40.13	−87.74	1	CP	Panarctic *D. pulex*	FF
Sportsman’s, IL	SPO	40.14	−87.44	2	CP	Panarctic *D. pulex*	FF
Long, IL	LON	40.13	−87.74	1	CP	Panarctic *D. pulex*	FF
Hill, MN	HIL	47.01	−93.36	1	CP	Panarctic *D. pulex*	FF
Glen, ON	GLE	45.08	−78.30	2	CP	Panarctic *D. pulex*	FF

**Table 2:** Components of reproductive isolation (*RI*_*i*_) and absolute (*AC*_*n*_) and relative (*RC*_*n*_) contributions to total reproductive isolation for the reproductive barriers (RIB) between *Daphnia pulex* and *Daphnia pulicaria* examined in this study. Components of reproductive isolation values are calculated based on modifications of [20], with C representing intrapopulation conspecific crosses, and vary from 0 (complete gene flow) to 1 (complete isolation). Components of reproductive isolation values are shown for *D. pulex* x *D. pulicaria* (px x pc), reciprocal *D. pulicaria* x *D. pulex* (pc x px), and the mean. Absolute and relative contributions are calculated based on [4], and in brackets showing total contributions of prezygotic and postzygotic isolating barriers. Total reproductive isolation is based on the sum of the absolute contributions of RIB. 95% confidence intervals are indicated in brackets

Reproductive barriers	Components of reproductive isolation (*RI*_*i*_*)*			Absolute contribution (*AC*_*n*_)	Relative contribution (*RC*_*n*_)
	Mean	px x pc	pc x px	Mean	Mean
Habitat	0.979 (± 0.029)	*–*	*–*	0.979	0.983
Temporal	0.313 (± 2.139)	–	–	0.007	0.007
Mating-Fertilization	0.134 (± 0.073)	0.192 (± 0.110)	0.060 (± 0.087)	0.002	0.002
Total Prezygotic				(0.988)	(0.992)
F1 Zygotic Mortality	0.134 (± 0.084)	0.137 (± 0.110)	0.132 (± 0.136)	0.002	0.002
F1 Hatching	0.366 (± 0.176)	0.296 (± 0.246)	0.446 (± 0.263)	0.004	0.004
F1 Survivorship	0.266 (± 0.147)	0.343 (± 0.224)	0.159 (± 0.183)	0.002	0.002
Total Postzygotic				(0.008)	(0.008)
Total Isolation				0.996	1.000

**Fig. 1:**
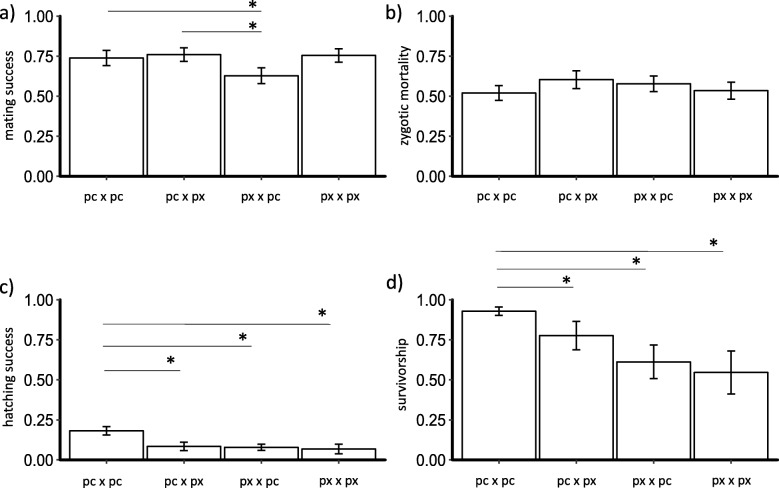
The mean proportion of non-ecological reproductive barriers of *Daphnia* across the four main cross categories: conspecific *Daphnia pulex* (px x px) and *Daphnia pulicaria* (pc x pc), and heterospecific *D. pulex* female x *D. pulicaria* male (px x pc) and the reciprocal cross *D. pulicaria* female x *D. pulex* male (pc x px). Shown in the panels are (**a**) mating-fertilization success, **b** F1 zygotic mortality, **c** F1 hatching success, and **d** F1 survivorship. Vertical bars are (±) standard errors. Asterisks (*) show significance (*p* < 0.05) between the different groups (shown in bars) based on a post-hoc Tukey test

**Fig. 2:**
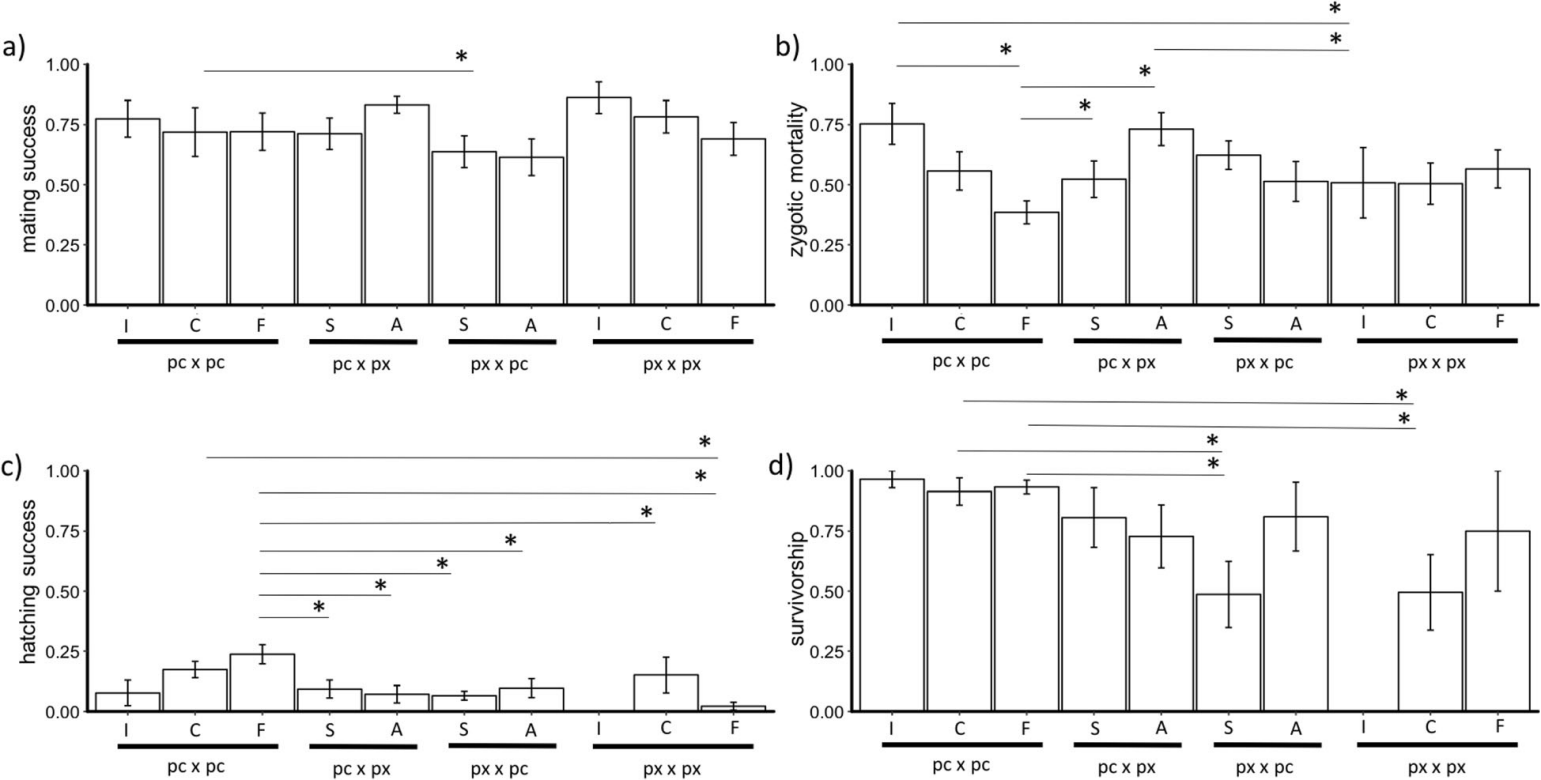
The mean proportion of non-ecological barriers of *Daphnia* across the different cross categories: **a** mating-fertilization success, **b** F1 zygotic mortality, **c** F1 hatching success, and **d** F1 survivorship. Each cross category is symbolized as: conspecific *Daphnia pulex* (px x px) and *Daphnia pulicaria* (pc x pc), divided into intrapopulation (I), geographically close (C) and geographically far (F) categories; and heterospecific *D. pulex* female x *D. pulicaria* male (px x pc) and its reciprocal cross, *D. pulicaria* female x *D. pulex* male (pc x px) divided into allopatric (A) and sympatric (S) groups. Each cross category is plotted with their respective (±) standard errors. In asterisks (*) show significance (*p* < 0.05) between the different groups (shown in bars) based on post-hoc Tukey test

Accounting for genotypic variation and temporal pseudoreplication, mating-fertilization success was slightly lower in heterospecific crosses (x̅ = 0.69) than in conspecific crosses (x̅ = 0.75). Heterospecific *Daphnia pulex* x *Daphnia pulicaria* had a significantly lower mating-fertilization success compared to *D. pulicaria* x *D. pulex* (post-hoc Tukey: *p* = 0.01, SE = 0.07; Fig. 1a). Conspecific (pc x pc and px x px) crosses had similar proportions of mating-fertilization success. For conspecific *D. pulex* crosses, geographically distant habitats had slightly lower mating-fertilization success compared with geographically close habitats. However, conspecific mating-fertilization success of *D. pulicaria* crosses was similar among geographically close and far habitats (Fig. 2a; Additional file 1: Table S4).

**Fig. 3:**
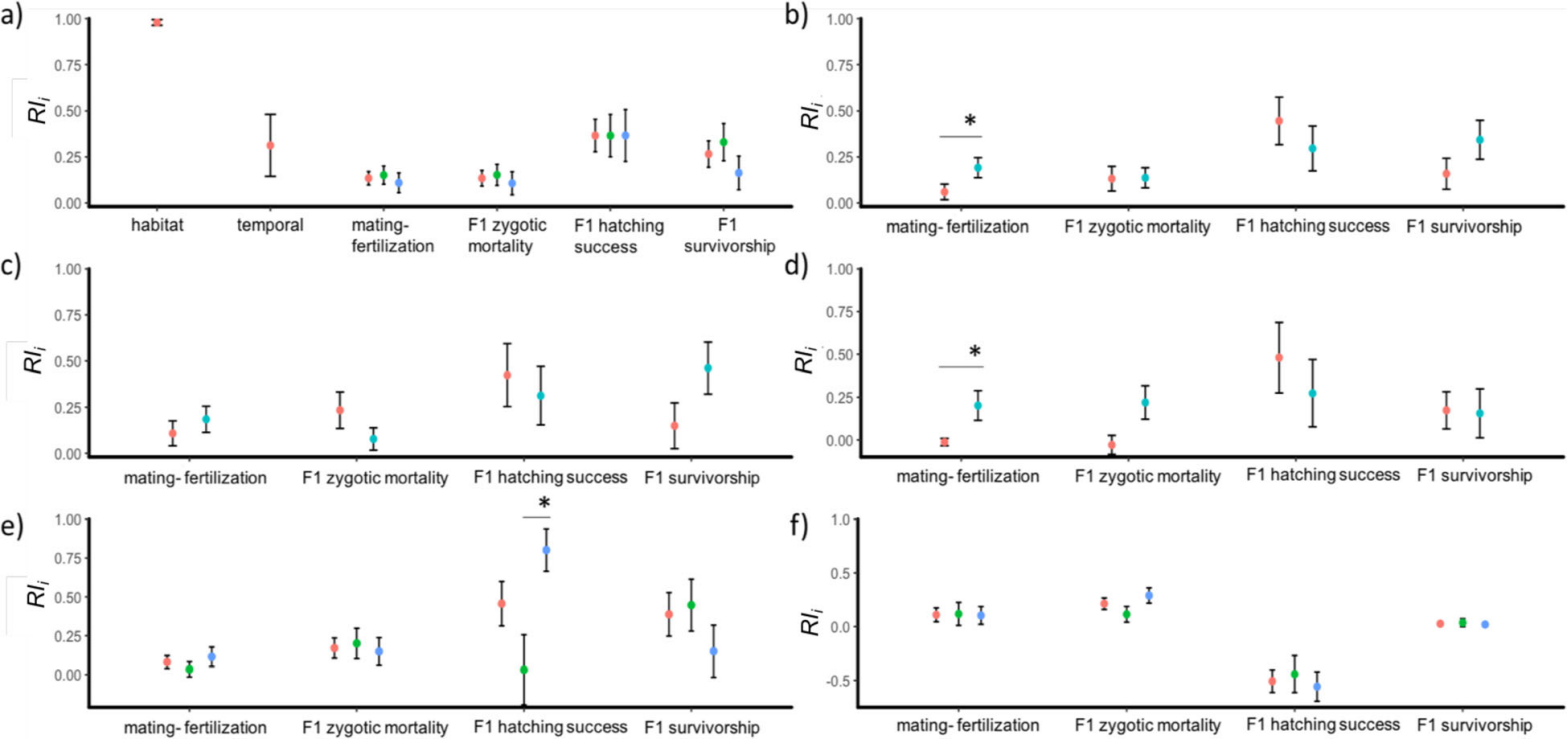
The independent contributions of reproductive isolation barriers (*RI*_*i*_) between *Daphnia pulex* and *Daphnia pulicaria*. **a** total mean reproductive barrier is shown in red, sympatric in green and allopatric populations in blue, **b** accounting for asymmetry of reproductive barriers for reciprocal crosses, reproductive barriers for *D. pulicaria* female crossed with *D. pulex* male in red*,* and *D. pulex* female crossed with *D. pulicaria* male in blue, **c** reproductive barriers accounting for asymmetry for sympatric populations, were sympatric *D. pulicaria* x *D. pulex* is in red and sympatric *D. pulex* x *D. pulicaria* in blue, **d** accounting for asymmetry for allopatric populations, where allopatric *D. pulicaria* x *D. pulex* is in red and allopatric *D. pulex* x *D. pulicaria* in blue. The reproductive barriers among conspecific populations of *D. pulex* (**e**) and *D. pulicaria* (**f**), where barrier strength of is shown for mean (red), geographically close (green) and geographically far (blue) crosses. In asterisks (*) showing significance (*p* < 0.05) between the different groups (shown in bars) based on Kruskal-Wallis one-way ANOVA test

The mean mating-fertilization RIB between the two species was 0.13, meaning that this barrier does not completely restrict gene flow between the two ecological species (Table 2). When comparing reciprocal crosses, we found this RIB to be asymmetrical, with *D. pulex* x *D. pulicaria* having a stronger RIB strength compared to *D. pulicaria* x *D. pulex*, and this pattern held in both allopatric and sympatric crosses. When examining conspecific crosses, mean mating-fertilization RIB for conspecifics was low (*RI*_*mating-fertilization*_ = 0.09), with *D. pulex* and *D. pulicaria* populations exhibiting similar strengths of mating-fertilization RIB. While in the case of *D. pulicaria*, geography does not appear to influence the barrier strength (Fig. 3f; Additional file 1: Table S5), for *D. pulex*, geographically far populations displayed a greater isolation than geographically close populations (Fig. 3e; Table 3).

**Table 3:** Components of reproductive isolation *(RI*_*i*_*)* among conspecific populations of *Daphnia pulex* (px) tested in this study. Components of reproductive isolation values are calculated based on modifications of [20], with C representing intrapopulation conspecific crosses, and vary from 0 (complete gene flow) to 1 (complete isolation). Components of reproductive isolation values are shown for geographically close crosses, geographically far crosses, and the mean. 95% confidence intervals are indicated in brackets

Reproductive barriers	Components of reproductive isolation		
	Mean	Geographically Close	Geographically Far
Mating-Fertilization	0.080 (± 0.085)	0.034 (± 0.110)	0.114 (± 0.133)
F1 Zygotic Mortality	0.170 (± 0.133)	0.200 (± 0.215)	0.148 (± 0.189)
F1 Hatching	0.456 (± 0.293)	0.031 (± 0.491)	0.801 (± 0.290)
F1 Survivorship	0.387 (± 0.316)	0.447 (± 0.395)	0.150 (± 2.130)

### Non-ecological postzygotic isolating barriers

From the experimental crosses, 1399 ephippia (2414 dormant embryos) were collected and stored in the dark for minimum of 1 month (~ 28 days) before hatching. After the hatching assay, each ephippia was opened to examine the dormant embryos for F1 zygotic mortality. Of the 2414 dormant embryos, 55.59% remained dormant, 34.71% were in the process of development but did not successfully hatch, and 9.69% had completed development and hatched (Additional file 1: Table S2). Taking into account genotypic variation and differences in storage time, F1 zygotic mortality was lower in conspecific (x̅ = 0.53) compared to heterospecific crosses (x̅ = 0.59; post-hoc Tukey test: *p* = 0.04, SE = 0.2). For heterospecific crosses, similar proportions of F1 zygotic mortality were found between reciprocal crosses (Fig. 1b). Lower proportion of F1 zygotic mortality was observed in sympatric crosses compared to allopatric crosses in *D. pulicaria* x *D. pulex* (Fig. 2b), while a discordant pattern was observed in reciprocal crosses (Fig. 2). Conspecific *D. pulicaria* and *D. pulex* had similar proportions of F1 zygotic mortality. For conspecific crosses of *D. pulicaria,* F1 zygotic mortality was lowest in geographically far crosses, and highest in intrapopulation crosses (post-hoc Tukey test: *p* < 0.01, SE = 0.5; Fig. 2b; Additional file 1: Table S4), whereas F1 zygotic mortality was similar for conspecific *D. pulex* irrespective of geographic distance (Fig. 2b).

The mean F1 zygotic mortality RIB between the two species is RI_*F1zygotic_mortality*_ = 0.13 (Table 2). Similar strengths of this RIB were found between reciprocal crosses. Examination of conspecific crosses found the mean F1 zygotic mortality RIB to be *RI*_*F1zygotic_morality*_ = 0.19. For *D. pulex*, geographically close crosses exhibited stronger barrier strength than geographically far crosses (Fig. 3e; Additional file 1: Table S5), while for *D. pulicaria*, geographically far crosses had a stronger barrier strength compare to close crosses (Fig. 3f; Table 3).

Of the 2414 embryos, 234 dormant embryos hatched (9.69% hatching success; Additional file 1: Table S2). About 95% of the individuals hatched during the first 7 days after exposure to a 12 h light: 12 h dark cycle at 18 °C; while 5% hatched after a second or third exposure. Heterospecific crosses displayed discordant patterns of F1 hatching success between allopatric and sympatric categories. *D. pulicaria* x *D. pulex* allopatric crosses had lower F1 hatching success compared to sympatric crosses, while F1 hatching success of sympatric *D. pulex* x *D. pulicaria* crosses was lower compared to allopatric crosses (Fig. 2c). Conspecific crosses had higher F1 hatching success compared to heterospecific crosses (post-hoc Tukey: *p* = 0.04, SE = 0.3). F1 hatching success was highest in conspecific *D. pulicaria* crosses compared to conspecific *D. pulex* (post-hoc Tukey: *p* < 0.001, SE = 0.4) and heterospecific crosses (*D. pulicaria* x *D. pulex:* post-hoc Tukey: p < 0.001, SE = 0.4; *D. pulex* x *D. pulicaria:* post-hoc Tukey: *p* = 0.008, SE = 0.4; Fig. 1c). For conspecific *D. pulex*, geographically close crosses had higher F1 hatching success than geographically far crosses, similar to conspecific *D. pulicaria* (Fig. 2c; Additional file 1: Table S4). Some ephippia from intrapopulation crosses in *D. pulicaria* did hatch, while none of the intrapopulation *D. pulex* ephippia hatched, suggesting that the cues used for hatching were likely better suited for one species than for the other.

The mean F1 hatching RIB between the two species was high (*RI*_*F1hatching*_ = 0.37; Table 2). Overall, this RIB barrier was symmetrical between sympatric and allopatric populations. When examining conspecific crosses, the F1 hatching RIB was very low for *D. pulicaria* but surprisingly high for *D. pulex* (*RI*_*F1hatching*_ = 0.46). For *D. pulex*, geographically far crosses had a stronger F1 hatching RIB compared to geographically close crosses (post-hoc Dunn’s test: *p* = 0.01, z = − 2.31; Fig. 3e; Table 3). The unexpectedly high level of *RI*_*F1hatching*_ could be a result of genetic incompatibilities between the two species as well as among distant populations. However, as we induced hatching using the same set of cues across all crosses, it is possible that some of the failures to hatch could be due to the wrong cue.

Out of the 234 individuals that hatched from ephippia, 177 individuals survived to adulthood and produced a first brood (75.64% survivorship). After taking into account the variability of the genotypes used to generate each cross, conspecific crosses show a higher mean F1 survivorship (x̅ = 0.81) to heterospecific crosses (x̅ = 0.68; post-hoc Tukey: *p* = 0.03, SE = 0.4). Similar proportions of F1 survivorship were found for allopatric and sympatric heterospecific crosses (Fig. 2d). Conspecific *D. pulicaria* showed higher F1 survivorship compared to conspecific *D. pulex* crosses (post-hoc Tukey: *p* < 0.001, SE = 0.6; Fig. 1d). Conspecific *D. pulicaria* crosses had similar F1 survivorship irrespective of geographic distance between habitats (Fig. 2d; Additional file 1: Table S4).

The mean F1 survivorship RIB between the two species was found to be *RI*_*F1survivorship*_ = 0.27 (Table 2). Similar strengths of this barrier were found in heterospecifics irrespective of direction and allopatry/ sympatry. The F1 survivorship RIB estimate for conspecific crosses was lower than in heterospecific crosses (*RI*_*F1survivorship*_ = 0.14). F1 survivorship RIB estimates were similar in *D. pulicaria* and *D. pulex* regardless of geography (Fig. 3ef; Table 3; Additional file 1: Table S5).

### Ecological prezygotic isolating barriers

We calculated habitat and temporal isolation between *D. pulex* and *D. pulicaria* using datasets from the literature. Based on ten population genetic datasets (Additional file 1: Table S3), we determined mean habitat isolation between *D. pulex* and *D. pulicaria* to be *RI*_*Habitat*_ = 0.979 (95% CI: ± 0.029; Table 2). We acknowledge that habitat isolation estimate could be inflated due to the limited *ldha* data readily available and our decision to exclude the SF genotypes (known to be obligately asexual in nature) in the calculation. We estimated the mean temporal isolation to be *RI*_*temporal*_ = 0.31 (95% CI: ± 2.14; Table 2) based on the dataset of [50, 60].

### Comparison of the strengths of reproductive barriers

Ecological barriers (habitat and temporal isolation) have had the greatest contribution to reproductive isolation compared to all nonecological prezygotic and postzygotic barriers (Table 2). We find that ecological prezygotic RIB contributes to 98.6% of total isolation. The prezygotic and postzygotic non-ecological barriers are much weaker contributing to only 0.2 and 0.8% respectively of the total isolation. Of the reproductive isolating barriers examined, none had the means to restrict gene flow between *D. pulex* and *D. pulicaria* completely. When comparing non-ecological isolating barriers, the greatest contribution to reproductive isolation was provided by F1 hatching success followed by F1 zygotic mortality and F1 survivorship.

## Discussion

While contemporary levels of gene flow estimated based on nuclear markers are relatively low between *Daphnia pulex* and *Daphnia pulicaria* [42, 59], likely due to strong ecological barriers, these species hybridize readily under laboratory conditions. We find that ecological barriers (habitat and temporal isolation) produced the largest contribution towards restricting gene flow. None of the non-ecological RIBs that we examined in this study (prezygotic or postzygotic) had the capability of completely restricting gene flow between *D. pulex* and *D. pulicaria*. We found asymmetry in the non-ecological prezygotic isolating barrier, where *D. pulex* x *D. pulicaria* crosses exhibited greater strength in mating-fertilization compared with the reciprocal *D. pulicaria* x *D. pulex* crosses, and this asymmetry was prevalent in allopatry. Interestingly, we found enhanced intrinsic postzygotic isolating barriers between geographically far populations of *D. pulex*, similar to patterns observed between the two species, suggesting emerging reproductive barriers within the currently recognized lineages.

### The role of prezygotic barriers to reproductive isolation

When examining prezygotic isolating barriers between *D. pulex* and *D. pulicaria*, we found that ecological RIB (habitat isolation and temporal isolation) had the greatest effect in restricting gene flow between the two species. As the two species inhabit distinct habitats and exhibit different life history traits as a result of such habitat differences, shifts in their timing of sexual reproduction could have evolved as a by-product of these differences. The induction of sexual reproduction depends mainly on photoperiod but also on food level or population density [61–63]. Ecological prezygotic isolating barriers have been previously hypothesized to be a major contributor in restricting gene flow between the two species [41, 59], and this observation is consistent with studies on other ecological species suggesting the importance of ecological divergence in promoting speciation [29, 64].

In the absence of ecological prezygotic barriers, *D. pulex* and *D. pulicaria* are capable of mating and producing viable dormant embryos. While our study did not distinguish between behavioural and mechanical isolation in these two species, previous studies point to the importance of these reproductive barriers in cladocerans [65–67], and observations in mating behaviour in *D. pulex* [68] and *D. pulicaria* [69] show some behavioural differences which could influence mating-fertilization success found between the two species in this study. Previous studies reported successful laboratory crosses between *D. pulex* females and *D. pulicaria* males [56]. Although we conducted successful crosses in both directions, comparisons between the reciprocal crosses indicate significantly lower mating-fertilization success in *D. pulex* females x *D. pulicaria* males (Fig. 1a), and therefore asymmetry in the mating-fertilization barrier (Fig. 3b). The efficiency of prezygotic reproductive barriers in restricting gene flow depends on the level and symmetry of historical gene flow between the sister species. Gene flow can be symmetrical or asymmetrical and this can influence the degree of symmetry in reproductive barriers. For example, flooding events from lakes to ponds often result in *D. pulicaria* colonizing pond habitats [41, 59]. In nature, the maternal parent of most hybrids is *D. pulex,* which suggests unidirectional hybridization between the two species [70]. In this scenario, the probability of *D. pulex* female residents encountering *D. pulicaria* males is higher than in the reciprocal direction. Unidirectional hybridization between closely related daphniid species appears to be common [71], as an example, experimental crosses of *Daphnia galeata* and *Daphnia cucullata* exhibited asymmetrical reproductive isolating barriers [72].

### The role of postzygotic barriers to reproductive isolation

While postzygotic isolating barriers may yield a smaller contribution towards total reproduction due to the sequential order of reproductive isolating barriers in the organisms’ life cycle, its independent contributions in restricting gene flow can play an important role in the absence of prior barriers. Furthermore, the addition of multiple reproductive barriers is necessary for complete isolation [17, 18]. Intrinsic postzygotic isolating barriers in association with a reduction in hybrid viability or fitness can be due to genetic incompatibilities such as Bateson-Dobzhansky-Muller (BDM) incompatibilities between the genomes of two species. According to the BDM model, incipient species that diverge in allopatry accumulate different mutational backgrounds, and during secondary contact, hybrids show a reduction in fitness compared to parental species due to negative epistatic interactions between the two genomes [73–75]. Hybrid performance can be further reduced in subsequent generations due to recombination events that break up epistatic interactions, facilitating reproductive isolation between the parental species.

In the absence of prezygotic isolating barriers, intrinsic postzygotic isolation appears to play a substantial role in restricting gene flow between *D. pulex* and *D. pulicaria*. Of the three intrinsic postzygotic barriers that we examined, F1 hatching had the greatest influence in restricting gene flow. In contrast, F1 survivorship was consistently high (Fig. 1d) and these barriers appear to have little influence on reproductive isolation (Fig. 3a). Overall, postzygotic isolating barriers displayed symmetry in their ability to restrict gene flow between *D. pulex* and *D. pulicaria*.

While postzygotic isolating barriers were thought to evolve slower in comparison to prezygotic isolating barriers [13], it appears that postzygotic isolation is important in restricting gene flow between these two species, which have diverged relatively recently (e.g. < 2 mya, based on mitochondrial markers [37]). Consistent with our results, [72] found low hatching and survivorship in experimental crosses between two closely related *Daphnia* species, *Daphnia cucullata* and *Daphnia galeata*. Similarly, intrinsic postzygotic isolating barriers play an important role for species that are currently in the process of ecological speciation [76].

### Emerging intraspecific reproductive isolation

One of our most unexpected findings was the very low hatching and survivorship experienced by conspecific populations of *D. pulex* crosses, which translates into a relatively high degree of postzygotic isolation (Table 3). This could be due to genetic incompatibilities in F1 hybrids during hatching and development, impeding survival to adulthood. Previous population genetics studies reveal an unexpectedly high level of genetic subdivision within *D. pulex* [59, 77]. Furthermore, theory suggests that fixation and accumulation of genetic incompatibilities occurs quickly in the absence of gene flow [78]. RIB studies of incipient species have also found the importance of intrinsic postzygotic isolation for diverging populations [34, 76, 79].

Records of successful hatching of dormant embryos has ranged anywhere from 2 to 65% for conspecific *D. pulex* crosses [80–83] and 20–100% for conspecific *D. pulicaria* crosses [84–86]. As hatching requirements vary between as well as within species [87, 88], and depend on environmental cues, it is possible that our experimental protocol was unable to reproduce the appropriate cues for hatching *D. pulex*. The timing of storage of ephippia in the dark was variable in our study (from 1 month to about 1.5 years); however, dormant embryos have been shown to maintain viability for long periods of time, 4 years [88] to 125 years [63]. This variation in storage time was accounted for in our generalized linear models and was found to have a negligible effect on hatching success (Additional file 2: Figure S2).

### The evolutionary mechanisms governing reproductive isolating barriers

The process of speciation is shaped by the evolutionary forces responsible for building and maintaining prezygotic and postzygotic reproductive barriers. The attention is often placed on how RIB emerge and a lot less is known about how barriers are maintained (but see [89]). Reinforcement of prezygotic barriers is thought to be a major evolutionary force for strengthening such barriers. In reinforcement, mating discrimination and mating preferences are enhanced in sympatric populations, where hybridization is most likely to occur, compared to allopatric populations [90–92]. Signatures of reinforcement have been found in a wide variety of taxa such as insects [93, 94], fish [95], birds [96] and mammals [97]. However, we found no evidence for stronger prezygotic isolation in sympatry than in allopatry.

Instead, we found evidence of asymmetrical reproductive isolating barriers for non-ecological prezygotic isolating barrier. Asymmetrical reproductive barriers have been found in a variety of organisms, and this pattern can occur in prezygotic [98, 99], postzygotic [100–102], or both types of barriers [10, 17, 103]. Asymmetry in prezygotic isolating barriers has been attributed to Kaneshiro’s hypothesis [104], where ancestral populations display stronger prezygotic barriers compared to derived populations due to relaxed mate choice mechanisms as a result of drift. In contrast, asymmetry in postzygotic isolation, called Darwin’s corollary, consists of a variety of BDM incompatibilities associated with uniparental inheritance [58, 105].

Additionally, we find evidence that postzygotic isolating mechanisms play an important role in restricting gene flow between intraspecific lineages of *D. pulex*, providing better understanding of the initial stages of speciation. This finding consolidated early speciation studies which found support for postzygotic isolating barriers among intraspecific lineages of the rainwater killifish *Lucania parva* (Cyprinodontiformes: Fundulidae) [24] and the spring peeper chorus frog *Pseudacris crucifer* (Anura: Hylidae) [34]. Other incipient plant species across various stages of the speciation continuum show the importance of postzygotic isolating barriers rather than prezygotic isolating barriers in restricting gene flow [22, 106, 107]. Collectively, these studies suggest that genetic incompatibilities accumulating between diverging populations could often mark the initial stages of speciation [108].

## Conclusions

This study examines prezygotic and postzygotic reproductive isolating barriers across the speciation continuum: from conspecific populations that are at the early stages of divergence to closely related species, within the young species complex of *Daphnia pulex*. We examine barriers that are emerging (the initial stage of speciation), as well as the barriers that are accumulating latter in the speciation process. We find that postzygotic isolating barriers appear to be responsible for the genetic subdivision reported within the *Daphnia pulex* lineage, suggestive of incipient speciation. We also find that ecological barriers are currently very strong and have the largest contribution towards restricting gene flow among the well-recognized ecological species. Thus, our results indicate that while non-ecological postzygotic isolating barriers were likely important during the initial stages of speciation, ecological, prezygotic isolating barriers are currently responsible for maintaining species boundaries. Our findings have implications for our understanding of the process of speciation revealing that current acting barriers are often not the same as early acting barriers and that the role of postzygotic isolation is likely underestimated, particularly when considering the very early stages of speciation.

## Methods

### *DAPHNIA* sampling, identification and culturing

To quantify non-ecological RIB, we established *Daphnia* clonal lineages from 13 populations (7 ponds and 6 lakes; Table 1, Additional file 2: Figure S1). About ten *Daphnia* individuals were isolated from each habitat and cultured in FLAMES media [109] at 18 °C with a 12 h light: 12 h dark cycle and fed twice a week with a mixture of *Pseudokirchneriella*, *Scenedesmus*, and *Ankistrodesmus*. All isolates were identified by morphology [47] and molecular markers using the protocol described by [59]. The mitochondrial NADH dehydrogenase subunit 5 (ND5) was amplified and sequenced to verify that all lineages belong to the *D. pulex* species complex. The *lactate dehydrogenase* A locus (*ldh*A) was amplified to differentiate the pond species (*D. pulex*, *ldh*A SS) from the lake species (*D. pulicaria*, *ldh*A FF). To confirm reproduction by cyclical parthenogenesis (sexual production of diapausing eggs), females were maintained in the absence of males and the deposition (or lack thereof) of dormant embryos in the ephippia were recorded based on the protocol from [110] (Table 1). Mature females carrying ephippia were selected from cultures. Males were isolated from cultures for at least 3 days prior to setting up the cross to ensure sexual maturity (see Additional file 1).

### Design of no-choice crosses

From the established clonal lines, no-choice crosses were set up to examine the absolute and relative contributions of non-ecological reproductive isolating barriers acting between *D. pulex* and *D. pulicaria.* We conducted conspecific crosses for *D. pulex* (px x px) and *D. pulicaria* (pc x pc) and heterospecific crosses (pc_♀_ x px_♂_; px_♀_ x pc_♂_; Additional file 1: Table S2). All crosses were replicated at least three times using individuals of the same genotype. We used identical female and male genotypes for the focal cross and the corresponding reverse cross. For each of the cross categories, we constructed at least two different crosses, using female and male genotypes originating from different habitats (e.g., px3_♀_ x pc4_♂_; Additional file 1: Table S2). Therefore, each cross category included individuals from a minimum of four habitats.

As species of the *D. pulex* complex are highly subdivided, showing strong genetic structure at fine geographical scale [38, 39, 57], we were interested in determining the level of genetic cohesion within each of the two recognized species. Thus, conspecific crosses were conducted among individuals originating from habitats that are geographically close (within 50 km) or far apart (greater than 500 km) (Additional file 1: Table S1). To estimate RIB among populations (conspecific crosses), as well as between species (heterospecific crosses), intrapopulation crosses were constructed as a baseline of performance (considered as C in our calculation for RIB), where individuals of distinct genotypes originating from the same habitat were crossed (e.g., px1_♀_ x px1_♂_).

As reproductive isolating barriers can be asymmetric in their strength depending on the direction of the cross, heterospecific crosses were conducted in reciprocal directions (px_♀_ x pc_♂_ and pc_♀_ x px_♂_). Moreover, given that reproductive isolating barriers can be influenced by the degree of gene flow occurring between interspecific gene pools, we constructed ‘allopatric’ and ‘sympatric’ crosses. We define allopatric populations as populations of *Daphnia* with low or restricted level of gene flow between lakes and ponds. We sampled *D. pulicaria* from lakes situated in regions containing only asexual (obligately parthenogenic) *D. pulex* clones [49]. Therefore, current gene flow between the two species is considered negligible. Sympatric populations were sampled from regions where we expect a high probability of gene flow between lakes and ponds (e.g., lakes with nearby ponds containing cyclically parthenogenic *D. pulex*).

## Estimating reproductive isolating barriers

### Non-ecological prezygotic isolating barriers

Each cross was assessed for the production of dormant embryos, which is a reflection of successful mating and fertilization. Females can revert back to parthenogenesis at any time during the experiment. Thus, the first time the female produced an amictic brood, the brood was removed and the cross was allowed to continue with the expectation that the female would revert back to the sexual phase. However, on the second amictic clutch, the cross was terminated. Each cross was maintained until a maximum of five ephippia were collected. Ephippia were opened under a Leica dissecting microscope. Each ephippium could have either 0, 1, or 2 dormant embryos. Absence of dormant embryos was interpreted as failure in mating and/ or fertilization. For each cross, the first ephippium produced by the female was opened and scored for dormant embryos, but not included in the calculation due to the possibility of previous fertilization prior to cross set up. Morphological analyses of daphniid females found no evidence of sperm storage receptacles [111, 112], and therefore we expect that females do not store sperm. All subsequent ephippia produced were included in the calculation. All opened ephippia with dormant embryos were stored at 4 °C in the dark to mimic wintering conditions and subsequently used for hatching.

### Non-ecological postzygotic isolating barriers

We examined three intrinsic postzygotic isolating barriers: F1 zygotic mortality, F1 hatching success, and F1 survivorship. For F1 zygotic mortality, we opened all ephippia at the end of the hatching assay to examine the appearance and quality of the dormant embryos. A score of 0 was assigned to embryos that began the process of development without successfully hatching, and a score of 1 was given to embryos that remained dormant and did not hatch. If fungal infections were observed, the embryos were categorized as inviable and given a score of 0. If dormant embryos successfully hatched, they were not included in the F1 zygotic mortality dataset. F1 zygotic mortality was calculated as the number of viable embryos over the total number of dormant embryos.

Hatching success was determined in a laboratory assay using ephippia from the experimental crosses that had been collected and stored at 4 °C in the dark. We set up the hatching assay during spring to promote favourable hatching conditions. We used natural spring water for rehydration to mimic natural freshwater habitats. Ephippia were exposed to a 12 h light: 12 h dark cycle at 18 °C for 7 days. If hatching did not occur after 7 days, ephippia were stored in the dark at 4 °C for 48 h before exposure to the same conditions for 2 months. Hatching ephippia in laboratory conditions is not without its caveats, as hatching is largely dependent on environmental cues [87, 113, 114]. Thus, embryos may not hatch because they did not detect the appropriate cues rather than having developmental defects. However, by exposing all cross categories to the same hatching cue, we standardize the performance of each cross category against the performance of the intrapopulation crosses. F1 hatching success was assessed by the number of dormant embryos that have hatched over the total number of dormant embryos that were recorded from the mating success dataset. Hatched neonates were placed in FLAMES media and observed for survivorship to adulthood. Survivorship was scored on a scale of 0 to 1 by assessing whether an individual was not able to reach adulthood (0) or reached adulthood and produced their first brood of clonal daughters (1). F1 survivorship was calculated as the number of individuals that survived over the total number of hatched embryos.

### Ecological prezygotic isolating barriers

To estimate habitat isolation (*RI*_*Habitat*_) between *D. pulex* and *D. pulicaria*, we searched the literature for population genetic surveys based on *ldh*A data (summarized in Additional file 1: Table S3). We calculated the number of instances of encountering both species in a particular habitat by recording whether homozygote SS (*D. pulex*) or FF (*D. pulicaria*) genotypes are present in both lake and pond habitats. We opted to omit heterozygote SF genotypes from the dataset, as SF genotypes found in nature are obligate parthenogenetic and not true F1 hybrids (see [115, 116]). We calculated habitat isolation for each study as follows:

$$ {RI}_{Habitat}=1-\frac{number\ of\ encounters\ in\ same\ habitat}{total\ number\ of\ encounters\ in\ same\ and\ different\ habitats} $$

This *RI* metric ranges from 0 (no restriction of gene flow) to 1 (complete restriction of gene flow). We took the mean of each calculated habitat isolation from the literature as *RI*_*Habitat*_ (Table 2; Additional file 1: Table S3)*.* We ran 10,000 bootstrap iterations to calculate the confidence intervals (Fig. 3a).

As facultative parthenogens, daphniids reproduce sexually during a few weeks of the year, although the exact timing of reproduction can vary among populations [77]. To estimate temporal isolation (*RI*_*Temporal*_) between the two species, we use temporal datasets from [50, 60], which report percent occurrence of sexually reproducing individuals of *D. pulex* and *D. pulicaria* under laboratory and natural settings. For species co-occurrence, we determined the area of overlap as the integral of the absolute differences in percent occurrence between the two species across the months/ photoperiods reported. We calculated temporal isolation between the two species as:

$$ {RI}_{Temporal}=1-\frac{\% overlap\ of\ individuals\  at\  sexual\ reproduction}{\% total\ individuals\  at\  sexual\ reproduction\ } $$

This *RI* metric ranges from 0 (no restriction of gene flow) to 1 (complete restriction of gene flow). We took the mean of these two datasets as an estimate of temporal isolation between the two species (Table 2). We estimated 95% confidence intervals by bootstrapping *RI*_i_ values using 10,000 bootstrap iterations (Fig. 3a).

### Quantifying the components of reproductive isolation

We calculated the strength of each reproductive isolating barrier (*RI*_*i*_) using methods modified from [20]:

$$ {RI}_i=1-2\left(\frac{H}{H+C}\right) $$

where H represents the frequency of successes for heterospecific or conspecific crosses, and C represents the frequency of successes for conspecific intrapopulation crosses. Considering the extreme levels of subdivision that occur within these two species [40, 77], and the uncertainty of whether the species exhibit genetic cohesion or are undergoing cryptic speciation, we define C as the mean frequency of successes of intrapopulation crosses in both *D. pulex* and *D. pulicaria*. Therefore, the *RI* metric ranges from 0 to 1, where 1 is the complete restriction of gene flow, and 0 indicates that there is no restriction of gene flow. We calculated the *RI*_*i*_ of each independent cross before taking the mean for each cross category to determine RIB. We also calculated 95% CI for mean RIB for each cross category.

### Statistical analyses

All our statistical analyses were done using R version 3.5.0 [117]. We implemented generalized linear mixed effects models (glmm) to account for random effects (e.g., differences in storage time for ephippia prior to hatching) in our datasets (R package *lme4* [118]). For each of our reproductive isolating barriers, we tested each response variable against the different cross categories as our dependent variables (e.g., survivorship ~ cross category). A post-hoc Tukey test (*multcomp* [119]) was implemented for multiple comparisons between the different cross categories.

In our mating-fertilization dataset, we constructed a poisson glmm with a log link function, where our response variable is the number of dormant embryos observed and our fixed variable is the cross category. We compared nested and non-nested models between the different cross categories; however, both glmms had similar Akaike Information Criterion (AIC) values. To account for temporal pseudoreplication, the number of trials was incorporated as a random effect (e.g. (trial|crossID)). Additionally, we accounted for differences in genotypes used in constructing each cross as a random effect.

To evaluate F1 zygotic mortality and F1 hatching, we constructed a binomial glmm with a logit link function, where the response variable for F1 zygotic mortality is the number of dormant/ defective embryos, and for F1 hatching is the number of hatched embryos, and the fixed variable is cross category. We compared nested and non-nested models of the different cross categories and found that non-nested models fitted better due to lower AIC values. As each ephippium collected during the experiment was stored immediately at 4 °C in the dark, there is a range of storage times, which may affect embryo viability. We accounted for the differences in storage time by incorporating it into the model as a random effect. We also considered differences in genotypes that were used to construct each cross as a random effect.

For the F1 survivorship dataset, we constructed a binomial glmm with a logit link function, where the response variable is survivorship and the fixed variable is cross category. We compared nested and non-nested models of the different cross categories and found that both models had similar fit to the dataset due to similar AIC values. We accounted for any differences in genotypes that were used to construct each cross as a random effect.

We were interested in comparing RIB estimates between sympatric and allopatric crosses and examining the symmetry of these barriers respective of the directionality of the cross. To examine the differences between these groups, a Kruskal-Wallis test and post-hoc Dunn’s test were performed.

### Absolute Contribution **(***AC*_*n*_**)** Towards Total Reproductive Isolation (*RI*_*Total*_)

Total reproductive isolation between *D. pulex* and *D. pulicaria* was inferred using two methods. First, we calculated the sequential strength of each barrier, or the “absolute contribution” (*AC*) [4], by ordering each barrier sequentially by its occurrence during the stages of its life history. The absolute contribution (*AC*_*n*_) of each RIB was calculated as the multiplicative function of its independent strength (*RI*_*i*_) and the amount of gene flow that remains unrestricted from its previous barriers that are acting earlier:

$$ {AC}_n={RI}_i\left(1-\sum \limits_{i=1}^{n-1}{AC}_i\right) $$

Total reproductive isolation is then calculated based on the sum of the absolute strengths of each barrier based on calculations from [4]. To determine the relative contribution (*RC*) of these isolating barriers have towards total reproductive isolation (*RI*_*Total*_), we use the equation from [4], where relative contribution (*RC*_*n*_) of each RIB is its absolute contribution (*AC*_*n*_) divided by total isolation (*RI*_*Total*_):

$$ {RC}_n=\frac{AC_n}{RI_{Total}} $$

## Supplementary information

**Additional file 1:** **Table S1.** Geographic distances (in kilometers) of lakes and ponds used in this study based on the Great Circle formula. **Table S2.** Total number of conspecific and heterospecific crosses set up within and among *Daphnia pulicaria* (pc) and *Daphnia pulex* (px) clones and the number of informative crosses that produced three consecutive ephippia (dormant embryos). For each cross category, the number of ephippia collected, the number of embryos that were hatched, and the number of individuals that survived to adulthood. **Table S3**. Habitat isolation estimates between *Daphnia pulex* and *Daphnia pulicaria* from previously published literature based on LDHA data. We calculated the probability of encounter in the same habitat by examining whether there are SS and FF genotypes present in the same habitat. For each study, we took the mean number of encounters found in the same habitat per study before calculating habitat isolation estimate. **Table S4.** Summary of mean proportions of mating-fertilization success, F1 zygotic mortality, F1 hatching success, and F1 survivorship (± standard error) for each *Daphnia pulex* (px) and *Daphnia pulicaria* (pc) cross category (N = number of unique crosses used in the analyses). **Table S5.** Components of reproductive isolation *(RI*_*i*_*)* between and among *Daphnia pulex* (px) and *Daphnia pulicaria* (pc). Components of reproductive isolation values are calculated based on modifications of [12], with C representing intrapopulation conspecific crosses, and vary from 0 (complete gene flow) to 1 (complete isolation). Components of reproductive isolation values are shown for heterospecific crosses, divided by sympatry and allopatry for *D. pulex* x *D. pulicaria* (px x pc), *D. pulicaria* x *D. pulex* (pc x px), and the mean, and for conspecific crosses for *D. pulex* (px x px) and *D. pulicaria* (pc x pc), divided into geographically close and far crosses, and the mean. 95% confidence intervals are indicated in brackets.

**Additional file 2:** **Figure S1.** Geographic distribution of North American *Daphnia pulex* (red) and *Daphnia pulicaria* (blue) and their naturally occurring hybrids (yellow). Enlarged map on the bottom right shows the sampling sites of ponds (red) and lakes (blue) used in this study. Sympatric habitats (circles) are identified as regions where there is high gene flow occurring between the two species, while allopatric habitats (squares) are identified as regions where there is a low potential for gene flow. **Figure S2.** The proportion of hatching success of F1 crosses plotted against the number of days in incubation in the dark at 4°C. A linear regression line (blue) is plotted, and shaded regions show confidence intervals

## Data Availability

Data available in the Dryad Digital Repository: 10.5061/dryad.n02v6wwsr.

## Abbreviations

AC: Absolute Contribution
AIC: Akaike Information Criterion
BDM: Bateson-Dobzhansky-Muller
C: Conspecific
H: Heterospecific
*ldhA*: lactate dehydrogenase subunit A
ND5: NADH dehydrogenase subunit 5
pc: *Daphnia pulicaria*
px: *Daphnia pulex*
RC: Relative contribution
RI: Reproductive isolation
RIB: Reproductive isolating barriers
*RI*_*i*_: Independent reproductive isolating barrier
*RI*_*TOTAL*_: Total reproductive isolation
