# Supplementary material for: The evolution of reproductive isolation in Daphnia
Source: BMC Evol Biol. 2019 Nov 27;19:216. doi: 10.1186/s12862-019-1542-9 (PMC6880586; doi:10.1186/s12862-019-1542-9)
Supplement: Supplementary file 1 — Additional file 1: Table S1. Geographic distances (in kilometers) of lakes and ponds used in this study based on the Great Circle formula. Table S2. Total number of conspecific and heterospecific crosses set up within and among Daphnia pulicaria (pc) and Daphnia pulex (px) clones and the number of informative crosses that produced three consecutive ephippia (dormant embryos). For each cross category, the number of ephippia collected, the number of embryos that were hatched, and the number of individuals that survived to adulthood. Table S3. Habitat isolation estimates between Daphnia pulex and Daphnia pulicaria from previously published literature based on LDHA data. We calculated the probability of encounter in the same habitat by examining whether there are SS and FF genotypes present in the same habitat. For each study, we took the mean number of encounters found in the same habitat per study before calculating habitat isolation estimate. Table S4. Summary of mean proportions of mating-fertilization success, F1 zygotic mortality, F1 hatching success, and F1 survivorship (± standard error) for each Daphnia pulex (px) and Daphnia pulicaria (pc) cross category (N = number of unique crosses used in the analyses). Table S5. Components of reproductive isolation (RIi) between and among Daphnia pulex (px) and Daphnia pulicaria (pc). Components of reproductive isolation values are calculated based on modifications of [12], with C representing intrapopulation conspecific crosses, and vary from 0 (complete gene flow) to 1 (complete isolation). Components of reproductive isolation values are shown for heterospecific crosses, divided by sympatry and allopatry for D. pulex x D. pulicaria (px x pc), D. pulicaria x D. pulex (pc x px), and the mean, and for conspecific crosses for D. pulex (px x px) and D. pulicaria (pc x pc), divided into geographically close and far crosses, and the mean. 95% confidence intervals are indicated in brackets. [file 12862_2019_1542_MOESM1_ESM.docx]

***Additional Files***

**Examining reproductive isolating barriers in *Daphnia***

**Authors:** Tiffany A. Chin, Carla E. Caceres, Melania E. Cristescu

**SUPPLEMENTARY TEXT**

**REPRODUCTION IN *DAPHNIA***

The common mode of reproduction in *Daphnia* is cyclical parthenogenesis, where most of the life cycle consists of amictic clonal propagation during favourable conditions, as it is usually the case for laboratory conditions, and sexual reproduction during stressful conditions (e.g. crowding, depletion of resources). During the sexual phase of their life cycle, female daphniids can either produce amictic diploid clonal males or haploid embryos. The haploid eggs are generated by the two ovaries from the female, and therefore the number of eggs produced for fertilization is often two. Successful fertilization of eggs through mating with diploid males results in the production of dormant embryos that are enclosed in an ephippium casing. In the presence of favourable hatching cues, dormant embryos will hatch into neonatal daphniids. In the case of unsuccessful fertilization, the ephippium produced will not contain dormant embryos [1].

**SAMPLING STRATEGY**

In order to identify pond and lake habitats with high or low levels of gene flow occurring between them, we generated a species distribution map of *Daphnia pulex* and *Daphnia pulicaria* using presence data from sampling sites reported from the literature. We did a search for each species using the ‘Web of Science’ database in 2014 to generate a list of publications with reported sampling sites. For each publication, we obtained the sampling site and GPS coordinates. For publications which did not report the GPS coordinates of the sampling site, we obtained the coordinates using Google Maps. However, where GPS coordinates were unavailable for the sampling site, the GPS of the nearest city or state was recorded instead. We also recorded whether the authors identified each species by morphological, ecological, or through genetic means. As *D. pulex* and *D. pulicaria* are morphologically almost indistinguishable from each other, sites that were only based on either morphological or ecological identification were removed from the dataset, and we only used sites for our distribution map where species were identified through both ecology and genetic means. A geographic distribution map of each species and the *D. pulex* x *D. pulicaria* OP hybrids found in nature was generated using ArcGIS using presence-only data (Figure S1).

**MOLECULAR IDENTIFICATION OF SPECIES**

Molecular identification based on the ND5 phylogeny revealed that all collected individuals belong to the panarctic *Daphnia pulex* lineage. For the diagnostic nuclear marker (LDH), daphniids were either homozygous for the S allele or the F allele, consistent with their pond (SS) and lake (FF) habitats. Sexuality tests confirmed the cyclical parthenogenesis (CP) reproductive strategy, and only CP clones were selected to set up the crosses (Table 1). Total number of crosses constructed and analyzed for this study is summarized in Table S2.

**LITERATURE CITED**

1. Innes DJ, Schwartz SS, Hebert PDN. 1986 Genotypic diversity and variation in mode of reproduction among populations in the *Daphnia pulex* group. *Heredity* **57**, 345–355. (doi: 10.1038/hdy.1986.134)
2. Hebert PDN, Schwartz SS, Ward RD, Finston TL. 1993 Macrogeographic patterns of breeding system variation in the *Daphnia pulex* group. I. Breeding systems of Canadian populations. *Heredity* **70**, 148-161. (doi:10.1038/hdy.1993.24)
3. Cerny M, Hebert PDN. 1993 Genetic diversity and breeding system variation in *Daphnia pulicaria* from North American lakes. *Heredity* **71**, 497-507. (doi:10.1038/hdy.1993.168)
4. Crease TJ, Lee S-K, Yu S-L, Spitze K, Lehman N, Lynch M. 1997 Allozyme and mtDNA variation in populations of the *Daphnia pulex* complex from both sides of the Rocky Mountains. *Heredity* **79**, 242-251. (doi:10.1038/hdy.1997.151)
5. Pfrender ME, Spitze K, Lehman N. 2000 Multi-locus genetic evidence for rapid ecologically based speciation in *Daphnia. Mol. Ecol*. **9**, 1717-1735. (doi:10.1111/j.0014-3820.2005.tb01754.x)
6. Dudycha JL. 2004. Mortality dynamics of *Daphnia* in contrasting habitats and their role in ecological divergence. *Fresh. Biol*. **49**, 505-514. (doi:10.1111/j.1365-2427.2004.01201.x)
7. Omilian AR, Lynch M. 2009 Patterns of interspecific DNA variation in the *Daphnia* nuclear genome. *Genetics* **182**, 325-336. (doi:10.1534/genetics.108.099549)
8. Heier CR, Dudycha JL. 2009 Ecological speciation in a cyclic parthnogen: sexual capability of experimental hybrids between *Daphnia pulex* and *Daphnia pulicaria*. *Limnol. Oceanogr*. **54**, 492-502. (doi:10.4319/lo.2009.54.2.0492)
9. Crease TJ, Floyd R, Cristescu ME, Innes D. 2011 Evolutionary factors affecting *Lactate dehydrogenase* A and B variation in the *Daphnia pulex* species complex. *BMC Evol. Biol*. **11**, 212. (doi:10.1186/1471-2148-11-212)
10. Cristescu ME, Constantin A, Bock DG, Cáceres CE, Crease TJ. 2012 Speciation with gene flow and the genetics of habitat transitions. *Mol. Ecol*. **21**, 1411-1422. (doi:10.1111/j.1365-294x.2011.05465.x)
11. Xu S, Innes DJ, Lynch M, Cristescu ME. 2013 The role of hybridization in the origin and spread of asexuality in *Daphnia*. *Mol. Ecol*. **22**, 4549-4561. (doi:10.1111/mec.12407)
12. Sobel JM, Chen GF. 2014 Unification of methods for estimating the strength of reproductive isolation. *Evolution* **68**, 1511-1522. (doi:10.1111/evo.12362)

**Tables**

**Table S1.** Geographic distances (in kilometers) of lakes and ponds used in this study based on the Great Circle formula.

|  | |  | | **ponds** |  |  |  |  |  |  | **lakes** |  |  |  |
| --- | --- | --- | --- | --- | --- | --- | --- | --- | --- | --- | --- | --- | --- | --- |
|  |  | CEN | DUM | BRI | TOP | DIS | SOL | STM | CLE | DEE | SPO | LON | HIL | GLE |
| **ponds** | CEN | - | 33.0 | 34.4 | 32.8 | 484.8 | 367.6 | 484.0 | 34.3 | 35.3 | 59.7 | 34.4 | 872.3 | 975.6 |
|  | DUM |  | - | 13.8 | 0.3 | 452.1 | 339.5 | 451.5 | 11.8 | 13.3 | 31.1 | 12.8 | 876.0 | 943.9 |
|  | BRI |  |  | - | 13.8 | 455.7 | 348.3 | 455.3 | 2.0 | 1.2 | 25.3 | 1.0 | 889.4 | 949.2 |
|  | TOP |  |  |  | - | 452.4 | 339.7 | 451.7 | 11.8 | 13.3 | 31.3 | 12.8 | 876.0 | 944.1 |
|  | DIS |  |  |  |  | - | 202.4 | 7.441 | 454.8 | 454.6 | 433.3 | 455.2 | 979.6 | 500.1 |
|  | SOL |  |  |  |  |  | - | 196.8 | 346.7 | 347.2 | 333.3 | 347.5 | 790.7 | 626.6 |
|  | STM |  |  |  |  |  |  | - | 454.3 | 454.2 | 433.2 | 454.8 | 971.3 | 497.5 |
| **lakes** | CLE |  |  |  |  |  |  |  | - | 1.6 | 25.34 | 1.0 | 887.5 | 948.0 |
|  | DEE |  |  |  |  |  |  |  |  | - | 24.4 | 0.9 | 889.2 | 948.0 |
|  | SPO |  |  |  |  |  |  |  |  |  | - | 25.3 | 900.2 | 928.3 |
|  | LON |  |  |  |  |  |  |  |  |  |  | - | 888.5 | 948.5 |
|  | HIL |  |  |  |  |  |  |  |  |  |  |  | - | 1183.2 |
|  | GLE |  |  |  |  |  |  |  |  |  |  |  |  | **-** |
|  |  |  |  |  |  |  |  |  |  |  |  |  |  |  |

**Table S2.** Total number of conspecific and heterospecific crosses set up within and among *Daphnia pulicaria* (pc) and *Daphnia pulex* (px) clones and the number of informative crosses that produced three consecutive ephippia (dormant embryos). For each cross category, the number of ephippia collected, the number of embryos that were hatched, and the number of individuals that survived to adulthood.

| Type of cross  (female x male) | Cross  Category | Number of crosses | Total number of unique crosses | Number of ephippia collected | Number of hatched embryos | Survivorship to adulthood |
| --- | --- | --- | --- | --- | --- | --- |
| pc x pc | overall | 140 | 33 | 383 (668) | 132 | 121 |
|  | intrapopulation | 40 | 10 | 100 (160) | 19 | 18 |
|  | close | 41 | 10 | 113 (199) | 38 | 35 |
|  | far | 59 | 13 | 170 (309) | 75 | 68 |
| px x px | overall | 134 | 32 | 344 (521) | 35 | 15 |
|  | intrapopulation | 26 | 6 | 60 (101) | 0 | NA |
|  | close | 50 | 11 | 128 (203) | 32 | 13 |
|  | far | 58 | 15 | 156 (217) | 3 | 2 |
| pc x px | overall | 108 | 30 | 348 (655) | 45 | 29 |
|  | sympatric | 62 | 18 | 171 (320) | 19 | 13 |
|  | allopatric | 46 | 12 | 177 (335) | 26 | 16 |
| px x pc | overall | 122 | 40 | 324 (570) | 22 | 12 |
|  | sympatric | 75 | 24 | 210 (373) | 10 | 5 |
|  | allopatric | 47 | 16 | 114 (197) | 12 | 7 |
| Total |  | 504 | 135 | 1399 (2414) | 234 | 177 |

**Table S3**. Habitat isolation estimates between *Daphnia pulex* and *Daphnia pulicaria* from previously published literature based on LDHA data. We calculated the probability of encounter in the same habitat by examining whether there are SS and FF genotypes present in the same habitat. For each study, we took the mean number of encounters found in the same habitat per study before calculating habitat isolation estimate.

| Habitat | Habitat Isolation Estimate | Literature |
| --- | --- | --- |
| ponds | 0.8411 | [2] |
| lakes | 0.9669 | [3] |
| lakes and ponds | 0.9998 | [4] |
| ponds | 0.9871 | [5] |
| lakes and ponds | 1 | [6] |
| lakes and ponds | 1 | [7] |
| lakes and ponds | 1 | [8] |
| lakes and ponds | 1 | [9] |
| lakes and ponds | 1 | [10] |
| lakes and ponds | 1 | [11] |
| Mean | 0.979 |  |

**Table S4.** Summary of mean proportions of mating-fertilization success, F1 zygotic mortality, F1 hatching success, and F1 survivorship (± standard error) for each *Daphnia pulex* (px) and *Daphnia pulicaria* (pc) cross category (N = number of unique crosses used in the analyses).

| Cross Category | Mating-fertilization success | F1 zygotic mortality | F1 hatching success | F1 survivorship |
| --- | --- | --- | --- | --- |
| pc x pc | (N = 35)  0.739 ± 0.047 | (N = 29)  0.520 ± 0.046 | (N = 29)  0.182 ± 0.027 | (N = 23)  0.929 ± 0.026 |
| intrapopulation | (N = 12)  0.774 ± 0.076 | (N = 6)  0.753 ± 0.086 | (N = 6)  0.077 ± 0.053 | (N = 2)  0.965 ± 0.035 |
| close | (N = 10)  0.719 ± 0.101 | (N = 10)  0.557 ± 0.080 | (N = 10)  0.173 ± 0.034 | (N = 9)  0.914 ± 0.056 |
| far | (N = 13)  0.721 ± 0.078 | (N = 13)  0.385 ± 0.048 | (N = 13)  0.237 ± 0.040 | (N = 12)  0.933 ± 0.028 |
| px x px | (N = 32)  0.754 ± 0.042 | (N = 33)  0.535 ± 0.053 | (N = 34)  0.068 ± 0.031 | (N = 10)  0.546 ± 0.134 |
| intrapopulation | (N = 6)  0.862 ± 0.065 | (N = 5)  0.508 ± 0.147 | (N = 5)  0.000 | n/a |
| close | (N = 11)  0.783 ± 0.067 | (N = 12)  0.504 ± 0.086 | (N = 13)  0.151 ± 0.074 | (N = 8)  0.495 ± 0.158 |
| far | (N = 15)  0.691 ± 0.068 | (N = 16)  0.566 ± 0.079 | (N = 16)  0.022 ± 0.016 | (N = 2)  0.750 ± 0.250 |
| pc x px | (N = 30)  0.760 ± 0.043 | (N = 36)  0.604 ± 0.056 | (N = 36)  0.084 ± 0.026 | (N = 13)  0.776 ± 0.088 |
| sympatric | (N = 18)  0.712 ± 0.065 | (N = 22)  0.523 ± 0.076 | (N = 22)  0.093 ± 0.037 | (N = 8)  0.806 ± 0.124 |
| allopatric | (N = 12)  0.833 ± 0.035 | (N = 14)  0.731 ± 0.068 | (N = 14)  0.071 ± 0.036 | (N = 5)  0.728 ± 0.131 |
| px x pc | (N = 39)  0.628 ± 0.049 | (N = 41)  0.314 ± 0.049 | (N = 41)  0.078 ± 0.019 | (N = 18)  0.612 ± 0.105 |
| sympatric | (N = 23)  0.637 ± 0.066 | (N = 24)  0.623 ± 0.059 | (N = 24)  0.065 ± 0.018 | (N = 11)  0.486 ± 0.138 |
| allopatric | (N = 16)  0.614 ± 0.076 | (N = 17)  0.514 ± 0.083 | (N = 17)  0.096 ± 0.039 | (N = 7)  0.810 ± 0.143 |

| Isolating barrier | Heterospecific | | | | | | | | | Conspecific | | | | | |
| --- | --- | --- | --- | --- | --- | --- | --- | --- | --- | --- | --- | --- | --- | --- | --- |
|  | Overall | | | Sympatry | | | Allopatry | | | px x px | | | pc x pc | | |
|  | Mean | px x pc | pc x px | Mean | px x pc | pc x px | Mean | px x pc | pc x px | Mean | Close | Far | Mean | Close | Far |
| Habitat | 0.979  (±0.029) | *---* | *---* | *---* | *---* | *---* | *---* | *---* | *---* | --- | --- | --- | --- | --- | --- |
| Temporal | 0.313  (±2.139) | *---* | *---* | *---* | *---* | *---* | *---* | *---* | *---* | --- | --- | --- | --- | --- | --- |
| Mating-fertilization | 0.134  (±0.073) | 0.192  (±0.110) | 0.060  (±0.087) | 0.151  (±0.100) | 0.185  (±0.147) | 0.108  (±0.143) | 0.110  (±0.110) | 0.201  (±0.185) | -0.013  (±0.047) | 0.080  (±0.085) | 0.034  (±0.110) | 0.114  (±0.133) | 0.109  (±0.133) | 0.117  (±0.242) | 0.103  (±0.180) |
| F1 zygotic mortality | 0.134  (±0.084) | 0.137  (±0.110) | 0.132  (±0.136) | 0.153  (±0.116) | 0.078  (±0.126) | 0.234  (±0.205) | 0.107  (±0.128) | 0.219  (±0.207) | -0.029  (±0.121) | 0.170  (±0.133) | 0.200  (±0.215) | 0.148  (±0.189) | 0.213  (±0.111) | 0.114  (±0.166) | 0.289  (±0.156) |
| F1 hatching success | 0.366  (±0.176) | 0.296  (±0.246) | 0.446  (±0.263) | 0.366  (±0.231) | 0.313  (±0.327) | 0.424  (±0.354) | 0.367  (±0.288) | 0.273  (±0.416) | 0.480  (±0.444) | 0.456  (±0.293) | 0.031  (±0.491) | 0.801  (±0.290) | -0.507  (±0.219) | -0.442  (±0.388) | -0.557  (±0.297) |
| F1 survivorship | 0.266  (±0.147) | 0.343  (±0.224) | 0.159  (±0.183) | 0.330  (±0.212) | 0.462  (±0.314) | 0.150  (±0.294) | 0.163  (±0.201) | 0.156  (0.351) | 0.173  (±0.302) | 0.387  (±0.316) | 0.447  (±0.395) | 0.150  (±2.130) | 0.026  (±0.037) | 0.036  (±0.086) | 0.019  (±0.035) |

**Table S5.** Components of reproductive isolation *(RI_i_)* between and among *Daphnia pulex* (px) and *Daphnia pulicaria* (pc). Components of reproductive isolation values are calculated based on modifications of [12], with C representing intrapopulation conspecific crosses, and vary from 0 (complete gene flow) to 1 (complete isolation). Components of reproductive isolation values are shown for heterospecific crosses, divided by sympatry and allopatry for *D. pulex* x *D. pulicaria* (px x pc), *D. pulicaria* x *D. pulex* (pc x px), and the mean, and for conspecific crosses for *D. pulex* (px x px) and *D. pulicaria* (pc x pc), divided into geographically close and far crosses, and the mean. 95% confidence intervals are indicated in brackets.
