# Supplementary material for: The evolution of reproductive isolation in Daphnia
Source: BMC Evol Biol. 2019 Nov 27;19:216. doi: 10.1186/s12862-019-1542-9 (PMC6880586; doi:10.1186/s12862-019-1542-9)
Supplement: Supplementary file 2 — Additional file 2: Figure S1. Geographic distribution of North American Daphnia pulex (red) and Daphnia pulicaria (blue) and their naturally occurring hybrids (yellow). Enlarged map on the bottom right shows the sampling sites of ponds (red) and lakes (blue) used in this study. Sympatric habitats (circles) are identified as regions where there is high gene flow occurring between the two species, while allopatric habitats (squares) are identified as regions where there is a low potential for gene flow. Figure S2. The proportion of hatching success of F1 crosses plotted against the number of days in incubation in the dark at 4°C. A linear regression line (blue) is plotted, and shaded regions show confidence intervals [file 12862_2019_1542_MOESM2_ESM.docx]

**Figure S1.** Geographic distribution of North American *Daphnia pulex* (red) and *Daphnia pulicaria* (blue) and their naturally occurring hybrids (yellow). Enlarged map on the bottom right shows the sampling sites of ponds (red) and lakes (blue) used in this study. Sympatric habitats (circles) are identified as regions where there is high gene flow occurring between the two species, while allopatric habitats (squares) are identified as regions where there is a low potential for gene flow.

**Figure S2**. The proportion of hatching success of F1 crosses plotted against the number of days in incubation in the dark at 4°C. A linear regression line (blue) is plotted, and shaded regions show confidence intervals.


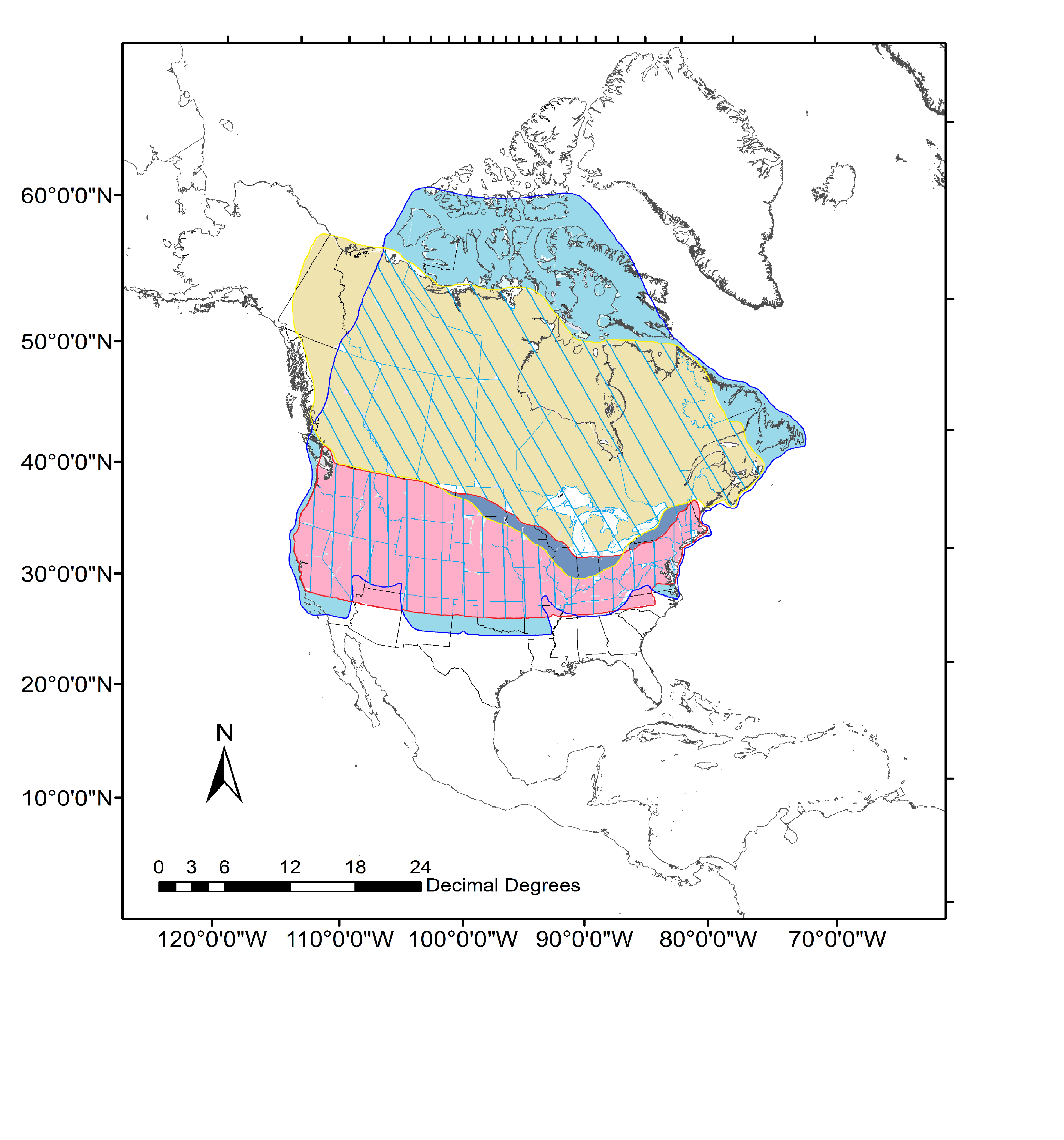

Proportion of hatching success

Number of days
